# Genome Analysis of *Microbacterium foliorum* Bacteriophage Annapurna

**DOI:** 10.17912/micropub.biology.001947

**Published:** 2026-03-31

**Authors:** Ashtyn Stock, Sarah Pritchard, Shelby Stone, Kinley Shelton, Emme Clowdus, Emily Doughtery, Sallie Hicks, Annabelle McDaniel, Kelsey Towle, Hannah Joseph, Kati Pourfarzib, Michael Baker, William Myers, Jadin Allen, Sydney Kruger, Kevin Perez, Jessie Holsombeck, Lori West, Dana Perry, Joseph Daft

**Affiliations:** 1 Natural Sciences, Lee University, Cleveland, Tennessee, United States

## Abstract

Bacteriophage Annapurna is a siphovirus discovered within a soil sample collected in North Georgia. Annapurna was isolated and amplified using the host *Microbacterium foliorum* prior to genome sequencing. Annapurna contains 84 predicted protein-coding genes encoded across a genome 56,247 base pairs in length. Based on gene content, Annapurna is assigned to actinobacteriophage cluster EF. Like other phages in the EF cluster, Annapurna appears to lack both integrase and repressor genes and is therefore predicted to be a virulent phage.

**Figure 1. Transmission electron micrograph of an Annapurna virion and plaque morphology:** A) Transmission electron micrograph of Annapurna showing a capsid diamter of 67-72nm and a 145-153nm tail (n=4), imaged at the University of Maryland Baltimore County. B) Plaque morphology of Annapurna plated with *Microbacterium foliorum.*

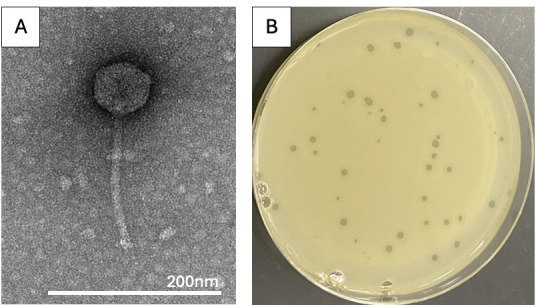



## Description

Due to the increase in antibiotic-resistant bacterial infections, there is a need for alternative treatment options. A promising avenue for treatment is the use of bacteriophages (Hatfull, 2022). In this study, *Microbacterium foliorum* NRRL B-24224, a common soil bacterium, was used as a host to isolate and purify a novel bacteriophage. The bacteriophage Annapurna was isolated from a dry soil sample collected from a cow pasture in Catoosa County, Georgia, USA (global positioning system [GPS] 34.56015N, 85.02109W) as previously described (Zorawik et al, 2024). Briefly, the soil sample was washed with peptone-yeast extract-calcium (PYCa) liquid media and the wash then filtered through a 0.22-µm filter. The filtrate was then plated in top agar with *M. foliorum* and plates incubated at 30˚C for 48 hours. A clear circular plaque, with a mean diameter of 1.90mm ± 0.78mm (n=20), was picked and designated Annapurna (Figure 1). Multiple rounds of plating were performed to purify Annapurna, after which a lysate was prepared and used for imaging and DNA extraction. Negative-stain (1% uranyl acetate) transmission electron microscopy (TEM) showed Annapurna to have a siphovirus morphology. (Figure 1).

Annapurna's DNA was extracted using the Promega Wizard DNA cleanup kit, prepared for sequencing using the NEB Ultra II FS kit, and sequenced at the University of Pittsburgh using Illumina NextSeq 1000 (XLEAP-P1 kit). The resulting 100-base reads were trimmed with cutadapt 4.7 (using the option: –nextseq-trim 30) (Martin, 2011) and filtered with skewer 0.2.2 (using the options: -q 20 -Q 30 -n -l 50) prior to assembly with Newbler v2.9 (Margulies et al., 2005) and assembly with 3,761-fold coverage checked for completeness with Consed v29 (Gordon et al., 1998). Annapurna's genome is 56,247 base pairs in length and has 63.5% GC content with circularly permuted ends. Based on gene content similarity equal to or greater than 35% to phages in the Actinobacteriophage database, PhagesDB, Annapurna is assigned to the EF cluster (Pope et al., 2017; Russell and Hatfull, 2017).

The genome of Annapurna was annotated utilizing Glimmer v3.02 and GeneMark v4.28 (Besemer and Borodovsky, 2005; Delcher, et al., 2007) to identify potential gene coding regions. These predictions were reviewed and refined in DNA Master v5.23.6 before functional predictions using amino acid sequences were assigned using HHpred MPI Bioinformatics Toolkit v3.3, using the PDB_mmCIF70, Pfam-v.37., NCBI Conserved Domains v3.21, and Uniprot SwissProt Viral70 databases (Söding et al., 2005; Pope and Jacobs-Sera, 2018). BLAST, using the Actinobacteriophage and NCBI non-redundant database, was used to confirm possible functions based on similarity with known sequences (Altschul et al., 1990). PhagesDB and Phamerator, with Actino_draft database v578, were used to compare Annapurna's genome with other phages in the EF cluster (Cresawn, 2011; Russell and Hatfull, 2016). DeepTMHMM v1.0.44 was used to identify transmembrane proteins while Aragon v1.2.41 and tRNAscan-SE v2.0.6 were used to identify tRNA sequences (Lowe and Eddy, 1997; Laslett and Canback, 2004). Default settings were used for all software during genome annotation. The annotation process revealed 84 predicted protein-coding genes, no tRNAs, and no tmRNAs. Of the protein-coding genes, 26 were assigned putative functions while 7 were identified as potential membrane proteins. The remaining genes code for proteins with no known function.

As with all 42 other annotated EF phages to-date, all predicted genes in Annapurna are transcribed unidirectionally. Additionally, there are two genes encoding for DnaE-like DNA polymerase alpha subunits and no integrase and repressor genes were identified, suggesting EF phages are unable to establish lysogeny (Jacobs-Sera, et al., 2020).

&nbsp;

**Nucleotide sequence accession numbers**

Annapurna is available at GenBank with Accession No. PV915857 and Sequence Read Archive (SRA) No. SRX29714288 .
